# Supplementary material for: Gotta Go Slow: Two Evolutionarily Distinct Annelids Retain a Common Hedgehog Pathway Composition, Outlining Its Pan-Bilaterian Core
Source: Int J Mol Sci. 2022 Nov 18;23(22):14312. doi: 10.3390/ijms232214312 (PMC9695228; doi:10.3390/ijms232214312)
Supplement: Supplementary file 1 [file ijms-23-14312-s001.zip › Supplementary figures and captions.pdf]

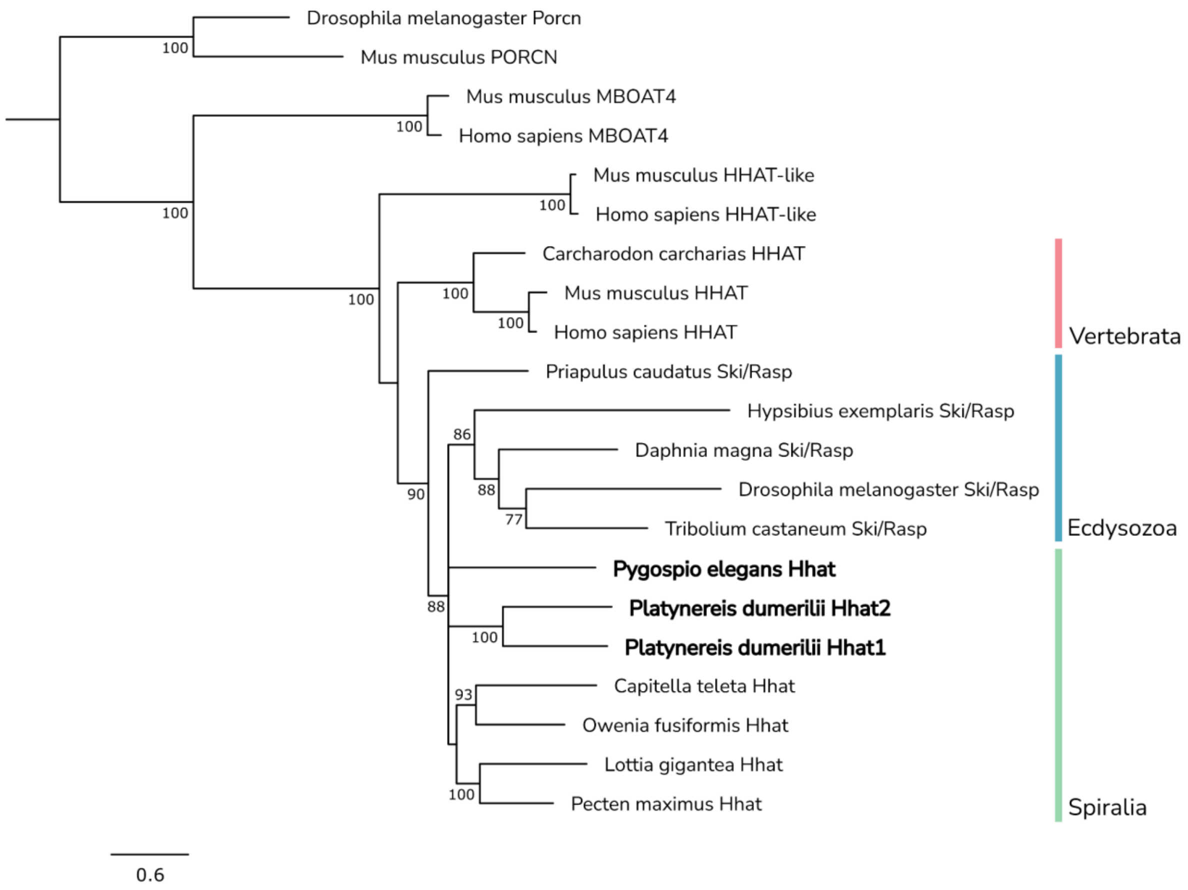

Figure S1. Phylogenetic analysis of Hhat proteins.

In *Platynereis*, we found two orthologues of Hhat proteins that show a sister relationship. In *Pygospio* and other annelid species, one Hhat orthologue was found; therefore, we suggest Pdum-Hhat1 and Pdum-Hhat2 may have arisen due to *Platynereis* specific gene duplication event. On the phylogenetic tree, identified annelids' proteins are associated with Ski/Rasp proteins from Ecdysozoa and HHATs from vertebrates. However, the analysis failed to resolve phylogenetic relationships among protostomes within the Skinny/Hhat clade. Two members of the MBOAT family were used as outgroups: Porcupine (Porcn) proteins from fly and mouse and ghrelin O-acyltransferases (MBOAT4) from human and mouse. Bayesian phylogenetic analysis was performed using amino acid sequences between terminal transmembrane helices. Numbers near branch nodes indicate Bayesian posterior probabilities. Bayesian posterior probability values less than 75 are not shown.

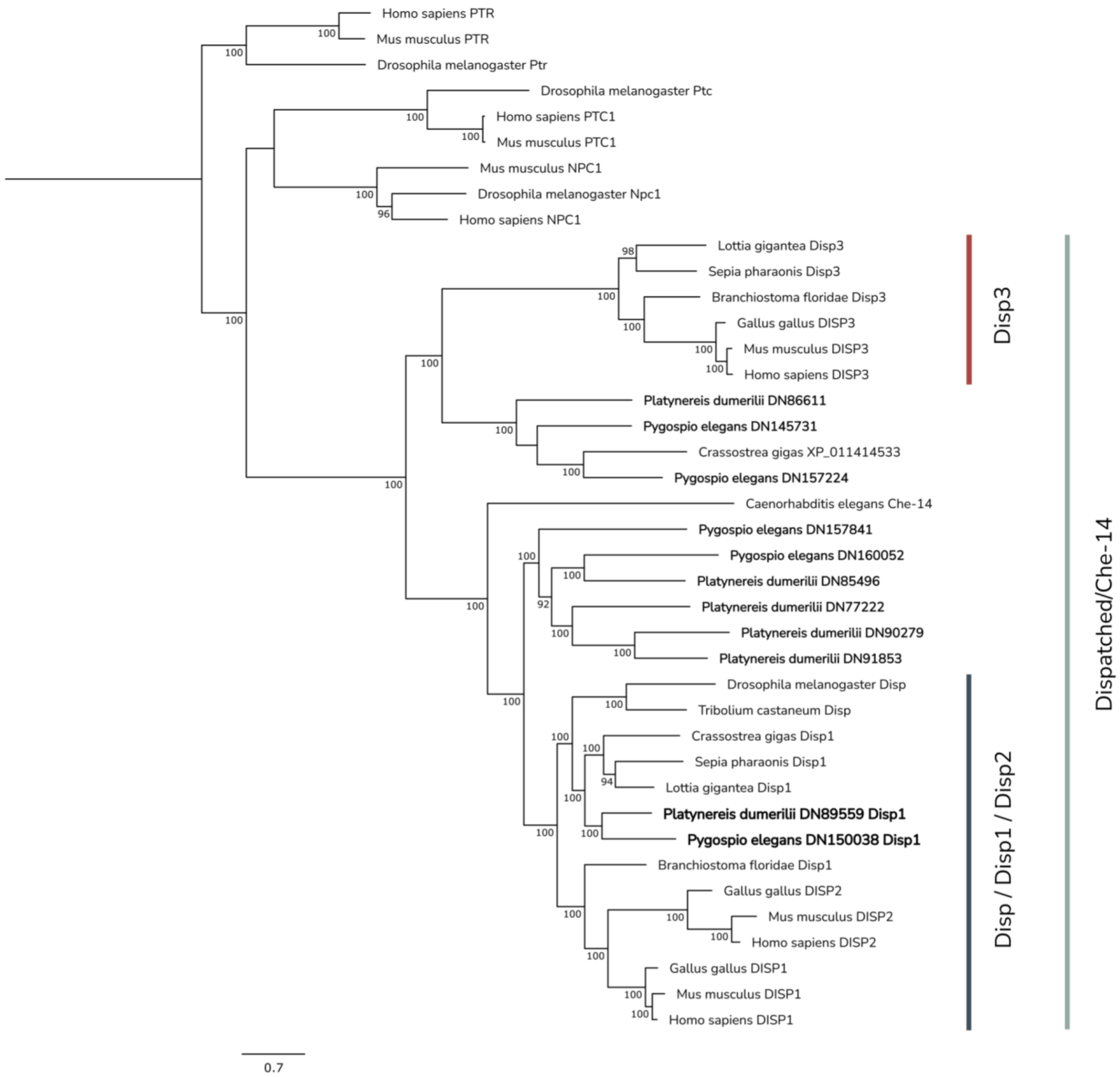

Figure S2. Phylogenetic analysis of metazoan Disp and Disp-related proteins.

In *Pygospio* and *Platynereis* transcriptomes, we found 11 proteins (highlighted with bold font) from the Dispatched/Che-14 family. Among them, two proteins fall into Disp/Disp1/Disp2 clade with known Disp proteins from fly and vertebrates. We named these proteins Pdum-Disp1 and Pele-Disp1 and took them for further analysis. The rest nine proteins fall into two distinct clades; one clade is in a sister relationship to Disp/Disp1/Disp2 clade, and the other forms a sister branch to metazoan Disp3 proteins. We did not find obvious Disp3 orthologs in both polychaetes. Contig IDs from a local database are written near *Pygospio* and *Platynereis* leaves. Patched-related (PTR) proteins from fly, mouse, and human were used as an outgroup. Numbers near branch nodes indicate Bayesian posterior probabilities. Bayesian posterior probability values less than 75 are not shown.

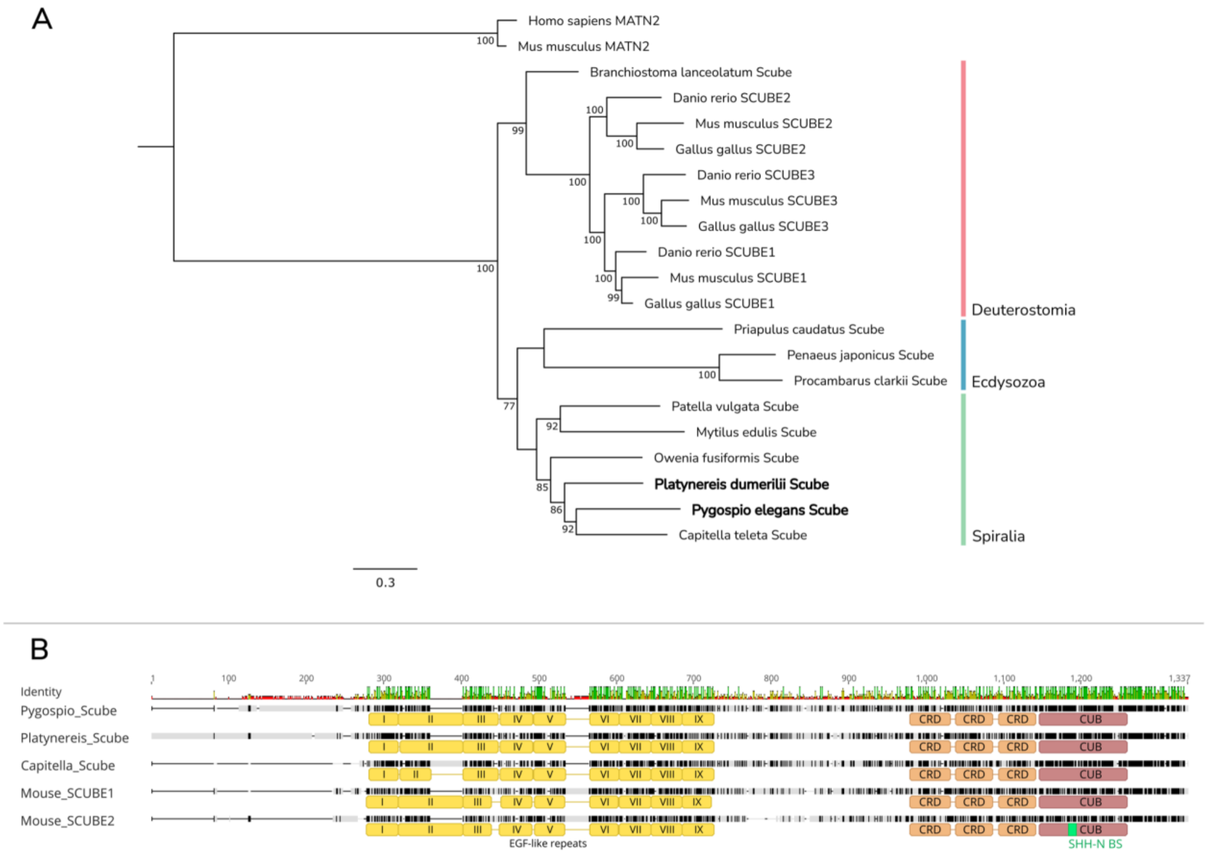

Figure S3. Phylogenetic analysis and multiple sequence alignment of Scube proteins.

A. While vertebrates possess three Scube proteins, we found one *Scube* gene in several invertebrate species, including *Platynereis* and *Pygopsio*. Results of the phylogenetic analysis suggest that Scube1, Scube2, and Scube3 are paralogues and could arise in vertebrate lineage due to the duplication event of a single *Scube* gene. Among ecdysozoans, crustaceans and *Priapulus caudatus* have one *Scube* gene, while *Drosophila* lacks orthologs of the Scube proteins. Numbers near branch nodes indicate Bayesian posterior probabilities. Bayesian posterior probability values less than 75 are not shown. B. Pele-Scube and Pdum-Scube are more similar to murine Scube1 (52% and 50% similarity respectively) than to Scube2 (50% and 46% similarity respectively). Murine Scube proteins consist of nine EGF-like repeats, three cysteine-rich domains, and one CUB (C1s-like) domain, which is essential for binding the cholesterol modification of SHH-N ligand. Pele-Scube and Pdum-Scube have the same domain structure. CRD, cysteine-rich domain; SHH-N BS, SHH-N binding site.

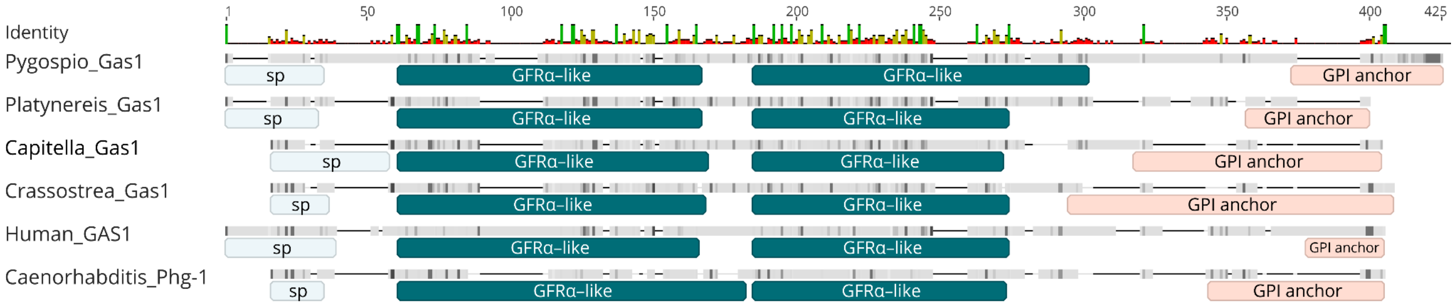

Figure S4. Multiple sequence alignment of Gas1 proteins. Gas1 is a glycosylphosphatidylinositol (GPI)3-anchored protein, which shows high structural similarity to the glial cell-derived neurotrophic factor receptor alphas (GFRαs). The human GAS1 domain structure is defined by two GFRα–like domains, an N-terminal signal peptide and a C-terminal GPI anchor. In Pele-Gas1 and Pdum-Gas1 sequences, we detected all these domains. GFRα-like, GFRα–like domain; sp, signal peptide.

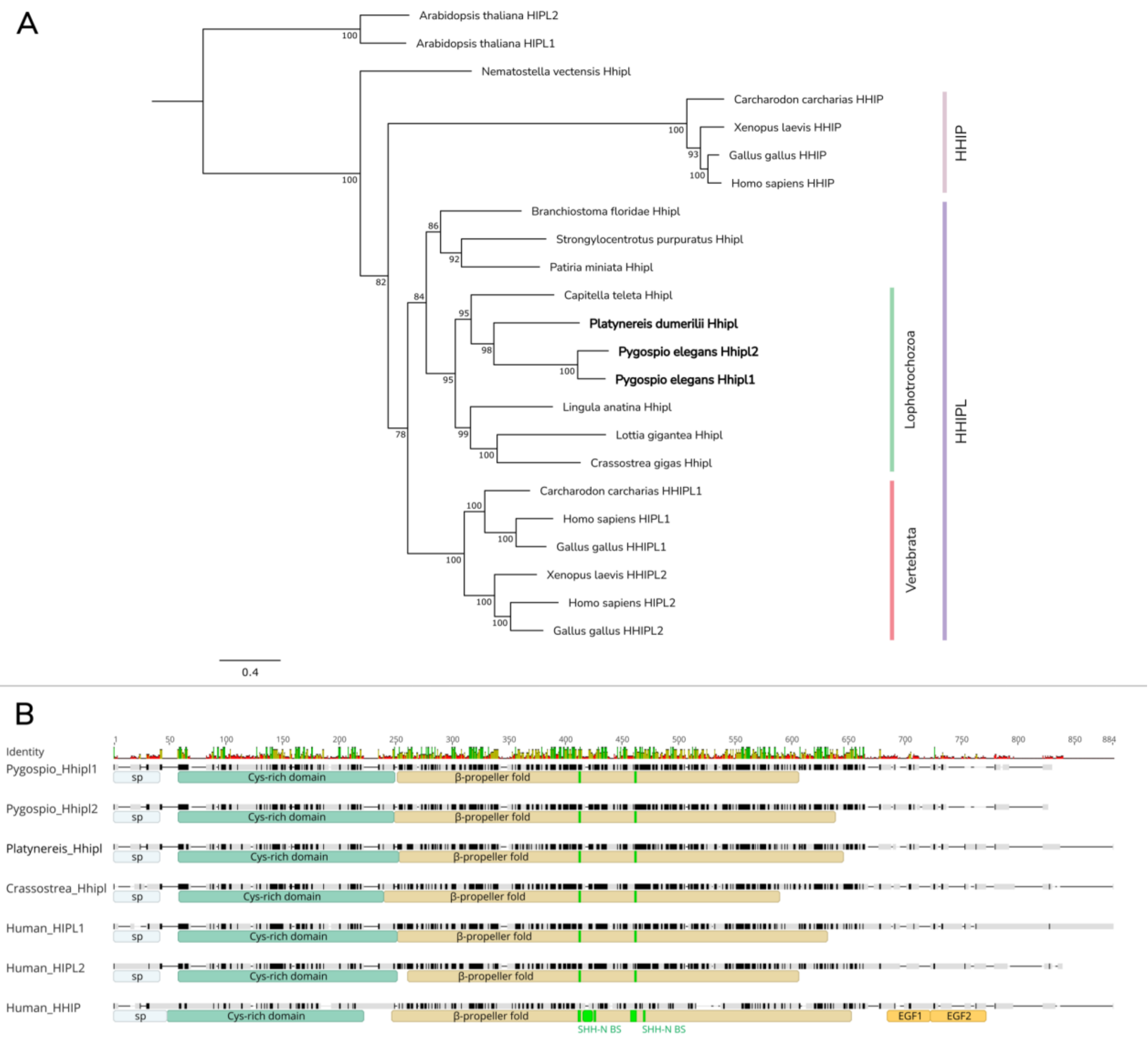

Figure S5. Phylogenetic analysis and multiple sequence alignment of Hhip family proteins.
A. Metazoan HHIP, HHIPL1 and HHIPL2 constitute HHIP family proteins, with HHIP being a vertebrate-specific inhibitor of the Hh signaling. HHIPL genes are present in Deuterostomia, Lophotrochozoa, and Cnidaria phyla and have not been found in Ecdysozoa yet. Most invertebrate species, including *Platynereis*, possess one HHIPL gene, but in the *Pygopsio* transcriptome, we discovered two sequences coding HHIPL orthologues. In vertebrates, there are also two HHIPL paralogues. However, considering the result of phylogenetic analysis, we speculate that duplication events in *Pygospio* and the common vertebrate ancestor were independent. Numbers near branch nodes indicate Bayesian posterior probabilities. Bayesian posterior probability values less than 75 are not shown.
B. Among HHIP proteins, the domain organization of human HHIP is better resolved. Human HHIP has an N-terminal cysteine-rich domain and a six-bladed b-propeller domain linked to two epidermal growth factor (EGF) repeat domains. Fourteen residues of the human HHIP are involved in the interaction with the SHH-N ligand (indicated by green bars). Spiralian and human HHIPL proteins have the same domain architecture; however, they do not possess a complete cluster which is required for the interaction with the Hh-N ligand. EGF1 and EGF2, epidermal growth factor repeat domains; sp, signal peptide; SHH-N BS, SHH-N binding site.

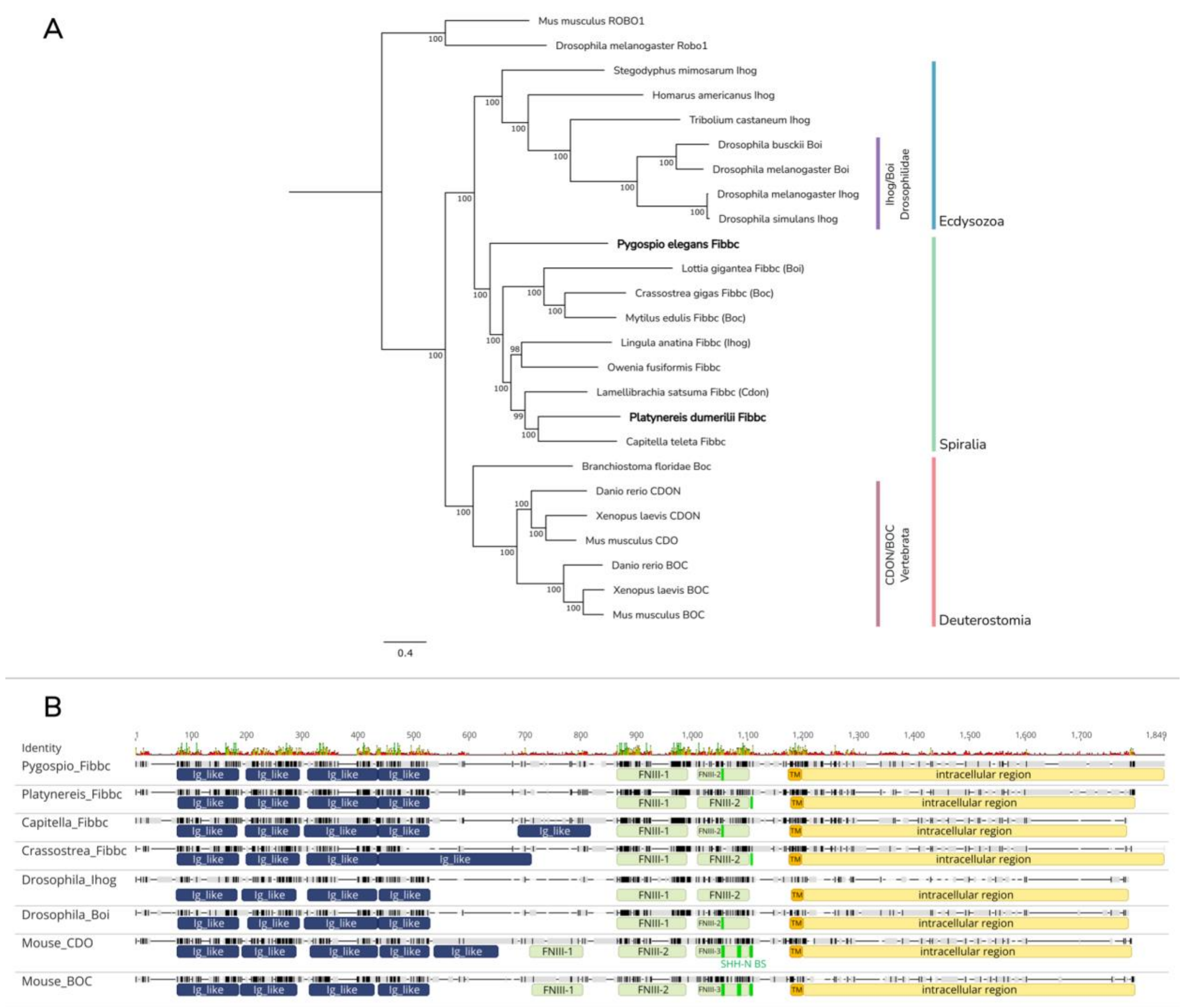

Figure S6. Phylogenetic analysis and multiple sequence alignment of Fibbc, Cdon/Boc, and Ihog/Boi proteins.

A. Vertebrate Cdon/Boc and *Drosophila* Ihog/Boi are transmembrane glycoproteins belonging to a subgroup of the Immunoglobulin (Ig) superfamily of cell adhesion molecules. Besides *Drosophila*, identifiable orthologues of Cdon/Boc and Ihog/Boi are present in other invertebrate bilaterian taxa. In both *Pygopsio* and *Platynereis* transcriptomes, we found one gene each encoding an orthologue to Cdon/Boc and Ihog/Boi. We named these proteins Pele-Fibbc (Father of Ihog/Boi/BOC/CDON) and Pdum-Fibbc, as they are equally unlike Cdon/Boc (16.7-18.6% identity, 26.6-31.0% similarity) and Ihog/Boi (15.1-17.9% identity, 25.4-29.8% similarity). In other spiralian species, we did not find two paralogues as well and named discovered proteins Fibbc indicating in parentheses available annotations from the NCBI database. The result of the phylogenetic analysis suggests that spiralians retained the single ancestral gene, which independently duplicated twice in vertebrates and drosophilids, leading to the origins of *Cdon/Boc* and *Ihog/Boi*. Robo axon-guidance receptors, another member of the Ig superfamily, are used as an outgroup. Numbers near branch nodes indicate Bayesian posterior probabilities. Bayesian posterior probability values less than 75 are not shown. B. Cdon/Boc and Ihog/Boi extracellular regions contain Ig-like domains and fibronectin type III-like (FNIII) repeats, followed by a single transmembrane domain and a divergent intracellular region. The ectodomain organization of Cdon/Boc and Ihog/Boi is not

the same: Cdon and Boc contain five and four Ig-like domains, respectively, and three FNIII repeats, while Ihog and Boi have four Ig-like domains and only two FNIII repeats. Furthermore, Cdon/Boc and Ihog/Boi bind Hh ligands with different modes. In murine CDO, eight sites of FNIII-3 repeat are involved in a calcium-dependent interaction with the SHH-N ligand (indicated by green bars). The binding between Ihog and the Hh-N ligand is heparin-dependent and involves the Ihog/Boi FNIII-1 repeat, which is not homologous to the Cdon/Boc FNIII-3. Having four Ig-like domains and two FNIII repeats, Pele-Fibbc and Pdum-Fibbc share similar domain organization with Ihog/Boi. Moreover, both proteins do not contain a complete cluster of residues required for interaction with the Hh-N ligand in a murine CDO manner, which suggests that an Ihog-like binding mode may occur in annelids. Ig-like, immunoglobulin-like domain; FNIII-(1-3), fibronectin type III-like (FNIII) repeats; TM, transmembrane domain.

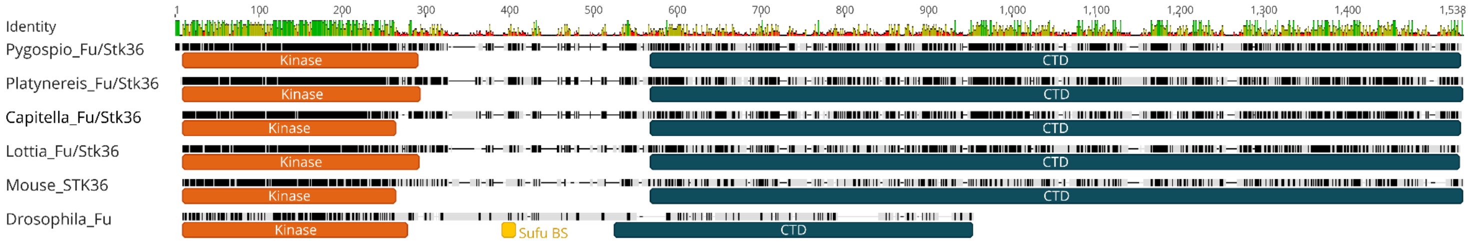

Figure S7. Multiple sequence alignment of Fu/STK36 proteins.

The *Drosophila fu* gene and its vertebrate homologue *STK36* encode serine-threonine kinases comprising an N-terminal kinase domain and a C-terminal regulatory domain (CTD), neither of which has been structurally characterized. In both proteins, CTDs are considerably less conserved than kinase domains, and in STK36, CTD is longer than the respective domain of Fu. Fu can bind to Sufu through the Sufu-binding site, which is necessary for efficient phosphorylation of Ci by Fu in Fu-dSufu-Ci ternary complex. Pele-Fu/Stk36 and Pdum-Fu/Stk36 share similar domain architecture, possessing the N-terminal kinase domain and the CTD. The length of the CTD and the absence of the Sufu-binding site indicate that the spiralian Fu/Stk36 amino acid sequence identities are closer to the mammalian STK36 than to *Drosophila* Fu. Kinase, kinase domain; CTD, C-terminal regulatory domain; Sufu BS, Sufu-binding site.

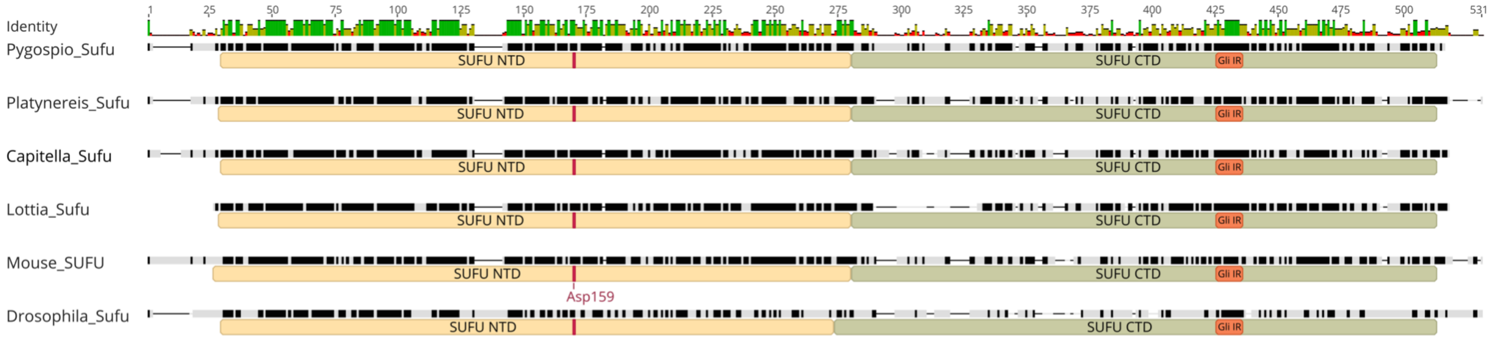

Figure S8. Multiple sequence alignment of Sufu proteins.

The *Sufu* genes encode cytoplasmic proteins with no apparent sequence homology to other proteins. The structure of a murine SUFU is the best to be characterized. SUFU contains two distinct domains: an N-terminal domain (NTD, Pfam: PF05076) and a C-terminal domain (CTD, Pfam: PF12470). Both domains are involved in binding and regulating GLI proteins. SUFU CTD contains a highly conserved region, which is required for binding the N-terminal end of GLI1; SUFU NTD binds the C-terminal tail of GLIs. Spiralian Sufu orthologues, including Pele-Sufu and Pdum-Sufu, also possess two distinct SUFU domains and the conserved Gli-interacting region. Furthermore, all Sufu orthologues have a conserved Asp159 residue (indicated by red bars) in which mutation results in the loss of Sufu activity. SUFU NTD, Sufu N-terminal domain; SUFU CTD, Sufu C-terminal domain; Gli IR, Gli-interacting region.

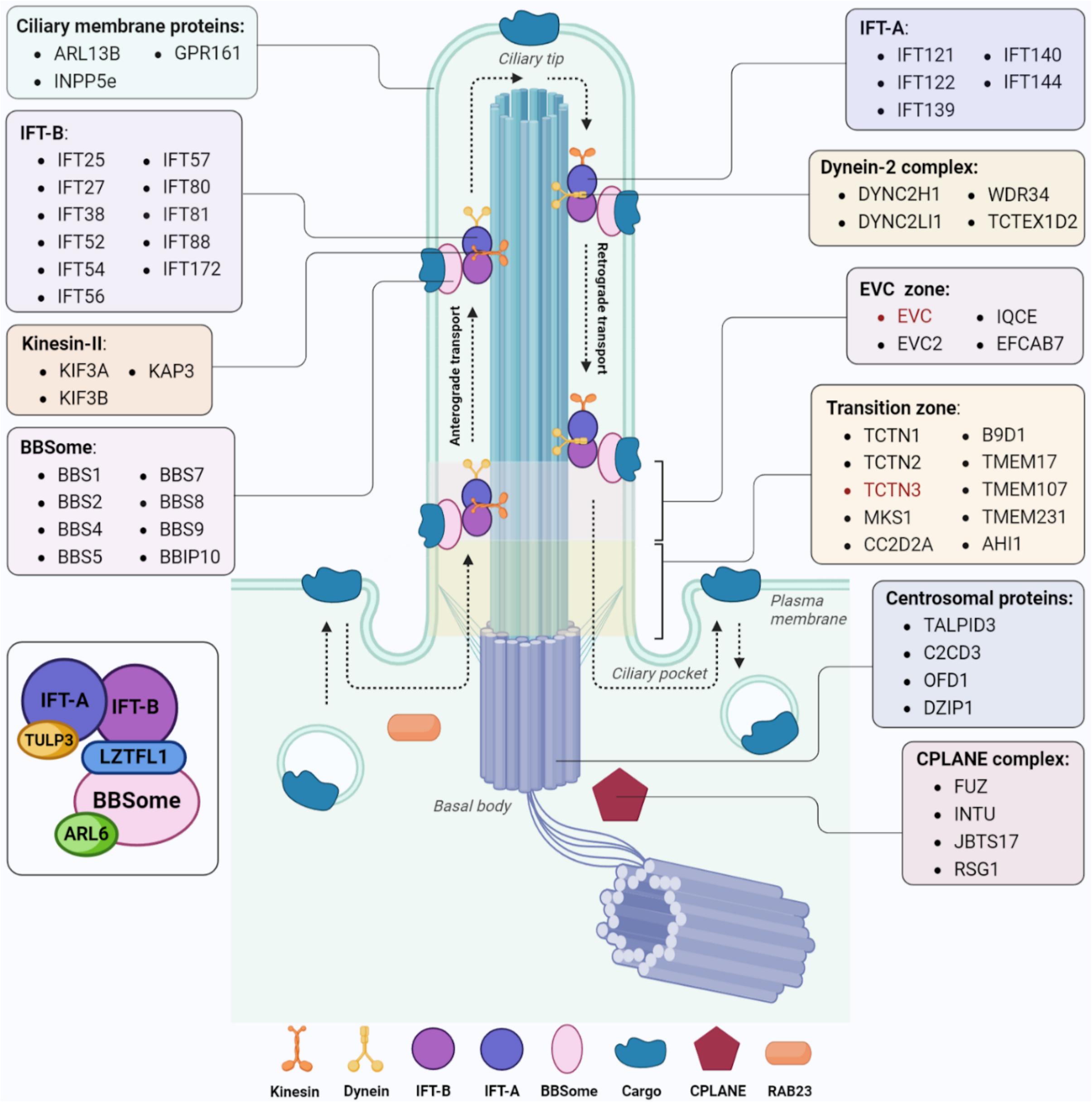

Figure S9. Graphic representation of primary cilium proteins which summarizes our results. The intraflagellar transport (IFT) apparatus mediates bidirectional protein trafficking within cilia, comprising anterograde IFT-B complex, powered by kinesin-II, and retrograde IFT-A complex, powered by the dynein-2 complex [9,109-111]. BBSome mainly functions as a cargo adapter, regulates protein trafficking to the ciliary membrane, regulates Hh signaling through SMO and PTC ciliary localization and removes GPCRs from cilia. Membrane recruitment of the BBSome requires the small GTPase ARL6, LZTFL1 mediates IFT/BBSome interactions [84,112-114,135]. Transition zone proteins are localized at the transition zone of primary cilia and required for Hh signaling by regulating the diffusion of transmembrane proteins between the cilia and plasma membranes [9,92,112,115-118]. EVC complex interacts with SMO and controls Hh signaling by GLI trafficking and SUFU/GLI dissociation [9,119,120]. ARL13B – ciliary membrane GTPase, regulates protein trafficking

and recruits INPP5E to the ciliary membrane. INPP5E - ciliary phosphatidylinositol phosphatase - mediates signaling through regulation of the phosphoinositol composition of the ciliary membrane [9,117,121-123]. GPR161 – negative regulator of signaling - is recruited by TULP3 and the IFT-A complex to primary cilia and acts as a regulator of the PKA-dependent processing of GLI. In presence of HH-N, it is removed from primary cilia and is internalized into recycling endosomes [9,93]. TULP3 interacts with the iIFT-A and is involved in the ciliary localization of transmembrane proteins (including ARL13B, INPP5E and GPR161) [9,124,125]. Centrosomal proteins C2CD3, TALPID3, OFD1, DZIP1 are required for ciliogenesis and Hh signaling regulation [9,118,126-128]. CPLANE (ciliogenesis and planar polarity effector) complex plays a key role in ciliogenesis and is involved in Hh signaling regulation [9,129,130]. RAB23 – small GTPase - regulates intracellular transport of membrane vesicles and together with SUFU prevents transport of GLI into the nucleus [131,132]. The genes which code proteins with the known role in signaling are in the frames. The genes which were not found in annelids are marked by red.
